# Debating the quality and performance of health systems at a global level is not enough, national debates are essential for progress

**DOI:** 10.1111/j.1365-3156.2008.02073.x

**Published:** 2008-04

**Authors:** Stephen Ntoburi, John Wagai, Grace Irimu, Mike English

**Affiliations:** 1 Kenya Medical Research Institute/Wellcome Trust Research ProgrammeNairobi, Kenya; 2 Department of Paediatrics and Child Health, University of NairobiNairobi, Kenya; 3 Department of Paediatrics, University of OxfordOxford, UK

**Keywords:** quality, health systems, performance

Assessing the quality of health care and in particular the part it plays in performance assessment of individuals, institutions and health systems is a topic of increasing interest in high-income settings (Elwyn *et al.* 2007; Heath *et al.* 2007; Majeed *et al.* 2007; Grol *et al.* 2008). What of low-income nations? Is it relevant to be concerned about quality? How might perspectives differ and are there some clear priorities? We make no claim to a representative ‘low-income country’ view. Our experience lies in the arena of child and newborn health in Kenya and it is from this vantage point we offer some thoughts. We do this with a hope of increasing awareness of, and debate around, quality and performance as major issues at country level in low-income settings.

From a global perspective the major focus of attention has been on assessing health systems’ performance. Many attributes considered within this framework, such as responsiveness, fairness (incorporating equity) or stewardship, overlap with those that might be considered indicative of the ‘quality’ of a health system (WHO 2000). At international levels quality and performance concerns reflect, among others, the global issues related to coverage of services (Bryce *et al.* 2006), availability and distribution of material and human resources (Hongoro & McPake 2004), and physical and financial barriers to access (Gwatkin *et al.* 2004). Areas in which the problems encountered in low-income settings make those of high-income countries appear inconsequential. However, these debates concentrate on cross-country comparisons or provide headline figures on global or regional problems. There is very little discussion, however, of quality and performance within a country's health system. We would like to adopt this perspective and suggest that country-level requirements have yet to receive the attention they deserve.

Does quality worry anyone? Understandably considerable investments are being made in improving coverage of and hence access to services. However, as coverage improves it is increasingly apparent that anticipated benefits will only be realized if services are of high quality. In Kenya quality is a major stated concern of the government and this prompted development of the Kenya Quality Model (Government of Kenya 2005). However, operationalizing this model remains difficult. As regards child and newborn health care, WHO have helped lead a still small but slowly growing interest in quality, at least from the perspective of small hospitals (Campbell *et al.* 2008). As with a handful of other low-income countries, the traditional Demographic and Health Surveys have now also been expanded to include a Service Provision Assessment (http://www.measuredhs.com/aboutsurveys/spa.cfm). However, these two laudable initiatives have limitations. The former provides an excellent opportunity for identifying key concerns and initiating debate and action, but it is not a measurement tool. The latter is a five-yearly situation analysis largely concentrating on structural components of quality. What more would we like to know?

In the traditional Donabedian approach, outcomes of the healthcare process are intuitively the most important if not the easiest to interpret. However, outcomes of value from the health systems perspective are varied, ranging from the fundamental, i.e. survival, through to the less immediately obvious such as the cost of providing a specific service. How well equipped are low-income countries to assess such outcomes? Unfortunately the answer is: not well equipped at all. Routine health management information systems (HMIS) rarely provide reliable information even on such basic indicators as the number of patients seen (Gething *et al.* 2007). In-hospital number of deaths and case fatality rates are rarely known accurately at facility level, let alone regional or national level. Information on surgical complication rates, nosocomial infection rates, frequency of medical errors or other potentially important medical outcomes is not available. Yet illustrative data suggest that potentially serious medical errors may occur on as many as 10% of occasions when even commonly used drugs are prescribed for sick children (English *et al.* 2004a). From a more managerial perspective, interest in clinic attendance rates and frequency of provision of medical, surgical or diagnostic procedures is largely restricted to their ability to generate local income through cost recovery. Using such data to inform local or national quality of care debates is rare. Understanding the performance of a particular set of clinical workers within a facility or of a facility itself is rarely possible.

The views of health system users are also an increasingly important outcome in judging the quality of healthcare provision in developed country settings. Are these views, felt, heard or acted upon in low-income settings? In the new healthcare (quasi-) markets of many industrialized countries, patient choice is, in theory, an indication of preference and thus a reflection of quality. The possibilities for choice in low-income countries are probably more varied than many imagine. In urban areas there has been a rapid and, some would say, largely unregulated expansion of the private sector. Larger, better-equipped facilities compete for the relatively small but financially important higher income groups who for many years have opted out of public healthcare systems. However, the greatest expansion has been in small, even single-provider, private clinics (Noor *et al.* 2004). Those belonging to the lower income groups prefer private clinics, because people have the general perception that the public sector performs poorly (Boller *et al.* 2003). Even in rural areas, where private facilities are fewer, patients often pay traditional practitioners for services than use ‘free’ public services. These choices may reflect preferences but we are aware of few attempts to examine satisfaction or similar aspects of quality at scale in our setting, although some institutions are now conducting ‘patient surveys’. However, satisfaction has been a topic of a reasonable body of research. It is hard to summarize this work succinctly and accurately, but it is clear that users in low-income settings are highly sensitive to quality issues. Absence of resources, inconvenient opening times, poor infrastructure, and staff attitudes and behaviour are all reported to be important aspects of quality even in poor, rural areas (Newman *et al.* 1998; Peterson *et al.* 2004). Interestingly users often express general satisfaction despite these concerns although their views may change depending on the technique, timing and setting of the process used for gathering information (Schneider & Palmer 2002).

Assessment of structural attributes of quality seems to be of little concern in many high-income settings – a basic and high standard of resource availability is usually assumed. Such assumptions would obviously be perilous in low-income settings. Yet where data on this aspect of quality are collected, they often reflect global agendas surrounding ‘essential services’. Lack of resources means that such data are often collected from samples of the health system to indicate the average level of provision. But while initially useful, the real need is to know, in every case, who does not have what. Indicators are often crude also. While activities such as essential drug-monitoring programmes initiated by the WHO and other efforts are slowly establishing standards for basic resource provision and evaluating systems on this basis, we are far from knowing what resources are available and where.

If the resources are available then achieving a worthwhile outcome depends on what we do as health workers – what we offer as a process of care. We will touch here on only a few issues pertinent to assessing the quality of the process of care limiting ourselves to clinical concerns. This ignores huge areas where quality is often poor, for example in the organization of services, respect for patient dignity or autonomy or rights of complaint, accountability or redress. Thinking about quality is, in our experience, often so alien to providers that they do not even realize that these topics and others are their concern. Potentially as damaging, however, is the situation in which providers and patients assume that the quality of care is adequate when in fact what is being offered is technically incorrect and occasionally dangerous.

For this reason, technical competence should retain a central concern in debates over the quality of care in low-income settings. In developed countries huge numbers of standards, guidelines and practice recommendations, based on evidence, define technical competence. Quality assessment and improvement activities in these countries often revolve around evaluating the extent to which practitioners follow this guidance, the degree of adherence being a major element in performance assessment. The WHO and others have developed and, with national governments, formally disseminated technical guidelines covering many areas of essential healthcare practice. However, these guidelines often do not reach practitioners, supervisors or even training institutions. We have a major ‘know’ gap before we even worry about the ‘know-do’ gap (WHO 2005). The result is that much basic practice is technically substandard even when resources are not limiting.

Low-income countries will therefore need to make huge strides in developing technical standards, disseminating these and monitoring their uptake if they are to tackle this ‘quality chasm’. The development of indicators for monitoring is in itself a science. Unfortunately it is a science that is largely undiscovered within public health systems in developing countries. Even if developed, the capacity for routine data collection, synthesis and analysis based on such indicators is often rudimentary. For this reason many ‘vertical’ programmes have established parallel reporting systems that allow them to assess quality indicators of relevance to their interest. Thus doses of immunizations given, numbers of tuberculosis or malaria cases receiving treatment or numbers of people receiving anti-retroviral drugs or family planning are monitored at facility level, often employing entirely different reporting systems. While serving a purpose, such parallel systems make it extremely difficult to derive any broader view on the quality of service provided by any one facility or provider unit, particularly as many programmes operate in only a subset of facilities. Sensitive and sensible monitoring for quality in the future will require a major investment in indicator development and information systems.

In turn this will require the development or strengthening of the building blocks of quality improvement that high-income countries take for granted. These include institutions to take on these roles and those guaranteeing professional standards, major improvements in health provider education, and a massive effort to build capacity and skills for lifelong, knowledge-based practice. It is also true that health provider attitudes, particularly among those in the medical profession, will need to change. Paternalism, lack of accountability and antagonism to change that threatens long-established modes of practice or behaviour are all still prevalent in many settings. Thus a culture of fear that precludes even activities as simple as audit to improve quality too often inhibits innovation, creative problem solving and true team working. Even where there is little actual antagonism to change as in developed countries, initial inertia may be profound.

It is clear that quality assessment and quality improvement have far to go in developing countries. It is often argued that solving resource limitations would transform the quality of care. This is in part because absence of resources is truly limiting on occasions and partly because of the link between inadequacies in resources, infrastructure and incomes and health worker motivation (Franco *et al.* 2003). Poorly motivated health workers seem unlikely to care too much about the quality of service they are providing. However, to label all healthcare settings and workers as unable to provide quality care does a disservice to the many institutions and people who perform to the best of their ability (English *et al.* 2004b). Assisting such people and facilities and encouraging others to follow their example should be the goal of quality improvement and thus of quality measurement. Solving essential resource constraints is thus necessary but not sufficient.

What should be our priorities then? As in high-income countries we will need to define quality and develop measurement approaches. Learning from higher income countries how to achieve this most efficiently as health systems develop seems prudent. Adopting uncritically developed country indicators, for example a current concern of policy makers with waiting times, may be dangerously distracting. Engaging with relevant professionals and professional bodies so that they lead quality improvement and performance enhancement approaches rather than challenging and obstructing them must also be initiated early. Functional health information systems will be needed to underpin most approaches and the profile of these departments must be enhanced while the nature of the data collected must reflect its ultimate use. In Kenya for example, even incomplete morbidity and mortality data are available for fewer than 50% of hospitals nationally and even when reported are un-interpretable because of systematic problems in the method of collection. Although the importance of health systems is increasingly appreciated, we are in danger of becoming solely pre-occupied with the global picture while we have made little headway in establishing what health systems are to do and how we will determine if they are achieving what is required within countries.
